# Digital phenotyping correlations in larger mental health samples: analysis and replication

**DOI:** 10.1192/bjo.2022.507

**Published:** 2022-06-03

**Authors:** Danielle Currey, John Torous

**Affiliations:** Division of Digital Psychiatry, Beth Israel Deaconess Medical Center, Harvard Medical School, Massachusetts, USA; Division of Digital Psychiatry, Beth Israel Deaconess Medical Center, Harvard Medical School, Massachusetts, USA

**Keywords:** Depressive disorders, anxiety disorders, mobile health, apps, digital health

## Abstract

**Background:**

Smartphones can facilitate patients completing surveys and collecting sensor data to gain insight into their mental health conditions. However, the utility of sensor data is still being explored. Prior studies have reported a wide range of correlations between passive data and survey scores.

**Aims:**

To explore correlations in a large data-set collected with the mindLAMP app. Additionally, we explored whether passive data features could be used in models to predict survey results.

**Method:**

Participants were asked to complete daily and weekly mental health surveys. After screening for data quality, our sample included 147 college student participants and 270 weeks of data. We examined correlations between six weekly surveys and 13 metrics derived from passive data features. Finally, we trained logistic regression models to predict survey scores from passive data with and without daily surveys.

**Results:**

Similar to other large studies, our correlations were lower than prior reports from smaller studies. We found that the most useful features came from GPS, call, and sleep duration data. Logistic regression models performed poorly with only passive data, but when daily survey scores were included, performance greatly increased.

**Conclusions:**

Although passive data alone may not provide enough information to predict survey scores, augmenting this data with short daily surveys can improve performance. Therefore, it may be that passive data can be used to refine survey score predictions and clinical utility may be derived from the combination of active and passive data.

The need to better quantify and understand the temporal dynamics and lived experiences of those with mental illness is critical to creating new accessible and digital treatments. Smartphones are a practical tool that are increasingly leveraged to collect both active data generated from participants interacting with the app, such as self-reported symptoms, and passive data collected in the background from meta-data and sensors, such as related behaviours, physiology and cognition. However, the potential to use information from these devices to predict mental health remains nascent, and pilot studies report varying estimates of the utility of this data. For example, smaller studies have found a range of different correlations between passive data features and survey scores.^1^ A study with one patient found correlations of 0.54 when using features extracted from microphone information, another study with five patients found correlations over 0.6 when using accelerometer features, and a study with 18 patients found correlations as high as 0.38 when using screen time data.^2–4^ In one of the largest studies to date, led by the company Verily, Nickels et al^5^ surveyed 415 participants over a 12-week study and examined correlations between numerous passive features, such as voice diary sentiment, location entropy and social app usage, and Patient Health Questionaire-9 (PHQ-9) survey scores. Except for voice diary sentiment and reported sleep duration, the study reported correlations of 0.1 or lower. Using an elastic net model, which is a penalised regression model that uses both L1 and L2 loss, and 34 features from the data, the authors obtained an area under the curve (AUC) of 0.656 for predicting mood. In another large study of 288 participants studying mood and anxiety, Meyerhoff et al^6^ employed a different approach, looking at correlations between changes in weekly survey scores and changes in passive data features. Focusing on GPS, call, text and app usage features, this study also reported low correlations similar to Nickels et al.^5^ Meyerhoff et al also separated participants into groups, using *k*-means clustering on the participants’ clinical scores, and found that some correlations were higher in groups exhibiting symptoms.^6^ In this work, we aim to explore correlations in a large data-set collected with the mindLAMP app from college student participants, to assess if we observe correlations of a similar magnitude to Nickels et al^5^ and Meyerhoff et al.^6^ In addition, we explore whether changing the group of participants that we use for analysis (such as by setting data-quality thresholds or by splitting into clinical groups) will allow us to identify more clinically meaningful correlations. Finally, we aim to test a classifier for predicting survey scores with passive and survey data, to assess whether passive data signals alone are enough to build predictive models or if survey data is necessary to provide a stronger signal.

## Method

### Data-set

Data were collected with the open-source mindLAMP app (this can be downloaded at https://docs.lamp.digital), developed by the Digital Psychiatry Lab at Beth Israel Deaconess Medical Center (BIDMC) and used by clinical and research teams around the world.^7–9^ mindLAMP is a smartphone app for iOS and Android that provides surveys, mindfulness audio and cognitive games to users. Additionally, mindLAMP can collect sensor data such as GPS, accelerometer and screen state from a participant's smartphone. A total of 695 college students were recruited to participate in the study between December 2020 and May 2021.

The authors assert that all procedures contributing to this work comply with the ethical standards of the relevant national and institutional committees on human experimentation and with the Helsinki Declaration of 1975, as revised in 2008. All procedures involving human patients were approved by the BIDMC Institutional Review Board (protocol 2020P000862), and all participants signed written informed consent. Through the app, participants were asked to complete short daily surveys and longer weekly surveys over a 4-week study period. The weekly surveys included the PHQ-9, Generalised Anxiety Disorder-7 (GAD-7), Perceived Stress Scale (PSS), UCLA Loneliness Scale, Pittsburgh Sleep Quality Index (PSQI) and Prodromal Questionaire-16 (PQ-16). Daily surveys consisted of a subset of the weekly survey questions. The questions can be found in Supplementary Appendix 1 available at https://doi.org/10.1192/bjo.2022.507. Passive data were also collected in the background. This included GPS, accelerometer, screen time, call and Bluetooth data.

### Features

We used raw passive data to compute features. From the GPS data, we computed home time, entropy, trip distance, location variance and unique location clusters. From the accelerometer, we estimated sleep duration. From call data, we took the number of incoming and outgoing calls, as well as the total duration of incoming and outgoing calls. From Bluetooth, we computed the nearby device count or the number of unique nearby Bluetooth devices detected by the smartphone. Finally, from device state data, we summed the total screen time. These features can be found on GitHub at https://github.com/BIDMCDigitalPsychiatry/LAMP-cortex.

As the quality of data can affect the quality of features, we set standards to reduce bias from missingness.^10^ We computed a metric of GPS data quality as the percentage of time that of one or more data points every minute was collected. Passive data on days where GPS frequency was <50% were excluded.

### Correlations between surveys and passive data

To explore how changes in passive data may be related to changes in active data, we aggregated scores for each week. Specifically, daily passive features and daily surveys were averaged over the 7 days up to and including the day on which the weekly survey was taken. We did not require a minimum number of the 7 days to have data to include that week of data. In total, this included 270 weeks of data for 147 participants. We were unable to collect Bluetooth or call data from Android smartphones, so our data-set included iOS devices exclusively. Correlations between the passive data features and weekly surveys were computed for different cohorts of the data. We compared correlations for the overall data-set with GPS data-quality constraints, without the data-quality constraints (which includes 190 participants and 358 weeks of data), for participants with slightly elevated depression and anxiety scores (PHQ-9 score >5 and GAD-7 score >4; including 79 participants and 121 weeks) and for participants with highly elevated depression scores (PHQ-9 score >16; including 15 participants and 19 weeks). Correlations were performed with the pearsonr function from the scipy.stats package (version 1.6.2), which can be found at https://docs.scipy.org/doc/scipy/reference/stats.html.^11^

### Logistic regression models

Logistic regression models were fit to the passive data features with and without the daily survey data. By imposing both an L1 and L2 penalty, we aimed to create more interpretable results and learn which features had predictive value by seeing which features had non-zero coefficients. Scores were computed by summing the scores for each question, which were a Likert scale from 0 (not at all) to 3 (nearly every day). Therefore, the maximum possible scores for each survey were 27 (PHQ-9), 21 (GAD-7), 30 (PSS), 60 (UCLA Loneliness Scale), 48 (PQ-16) and 27 (PSQI). For the overall weekly models, scores were considered to be high if they were greater than a set threshold based on the clinical literature. For the PHQ-9, GAD-7 and PSS, we used a threshold of 10; for the PSQI, a threshold of 5 was used; for the PQ-16 a threshold of 6 was used and for UCLA Loneliness Scale, a threshold of 20 was used.^12–15^ We also fit models to the individual questions of the weekly surveys. We considered scores of 2 or 3 (on a range of 0–3) to be elevated. Some questions had limited data in the elevated group (for example, only ten participants reported severe and active thoughts of suicide), which prevented the models from converging. In this case, the AUC was assumed to be 0.5. Five-fold cross-validation was performed. The Scikit-Learn LogisticRegression model was used with an l1_ratio of 0.5.^16^ Class weights were balanced and all input features were standardised.

## Results

**Fig. 1:**
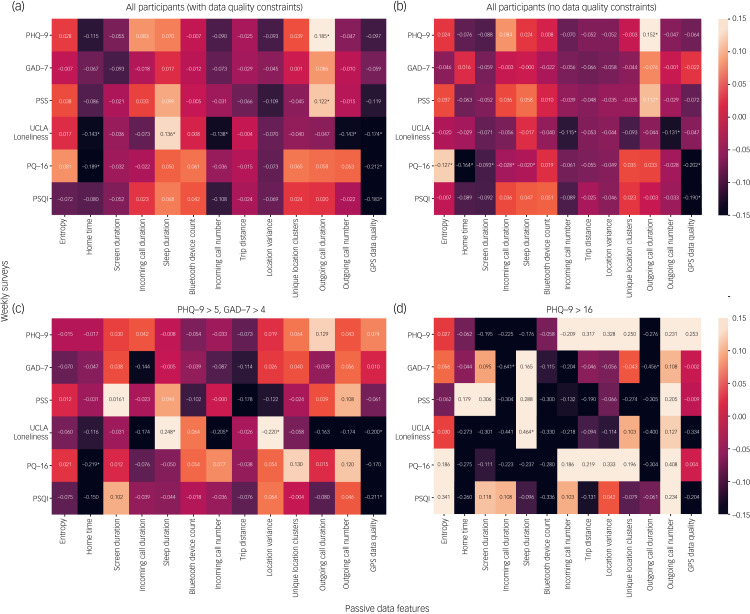
Weekly correlations between surveys and passive data features. (a) Overall correlations with passive features including only participants that met the data quality constraints. (b) Correlations if data quality is not considered. (c) Correlations for weeks where patient PHQ-9 scores were >5 and GAD-7 scores were >4. (d) Correlations for weeks where PHQ-9 scores were >16. Correlations with *P* < 0.05 are marked with an asterisk. As these correlations are small, the heatmap has been scaled to –0.15 to 0.15, to show the differences in the correlations. GAD-7, Generalised Anxiety Disorder-7; PHQ-9, Patient Health Questionnaire-9; PQ-16, Prodromal Questionnaire-16; PSQI, Pittsburgh Sleep Quality Index; PSS, Perceived Stress Scale.

Figure 1 shows correlations differ based on the subset of data used. Figure 1(a) shows correlations from all participants with the data-quality requirements as described above. Figure 1(b) shows the correlations without data-quality requirements, including participants with poor data quality. Figures 1(c) and 1(d) show the correlations for the subset of patients with higher PHQ-9 and GAD-7 scores.

There are several correlations that persist across the different groups. Home time is inversely correlated with PQ-16 scores in all groups except for the very high PHQ-9 group. Sleep duration and UCLA Loneliness Scale scores are positively correlated for all but the group without data-quality constraints, which includes participants with poor data quality. The number of incoming calls is also negatively correlated with UCLA Loneliness Scale in all groups except for the very high PHQ-9 group. GPS data quality is negatively correlated with PSQI scores for all but the highest PHQ-9 group. Additionally, outgoing call duration is correlated with PSS and PHQ-9 scores for the groups containing both symptomatic and asymptomatic participants, but not in groups with only higher PHQ-9 scores.

Despite these similarities, we also see differences across the groups. For example, a correlation between entropy and PQ-16 is seen in the poor data-quality group, but not in any other group. Moreover, there is a correlation between outgoing call duration in Figures 1(a) and 1(b) but not in Figures 1(c) and 1(d).

**Fig. 2:**
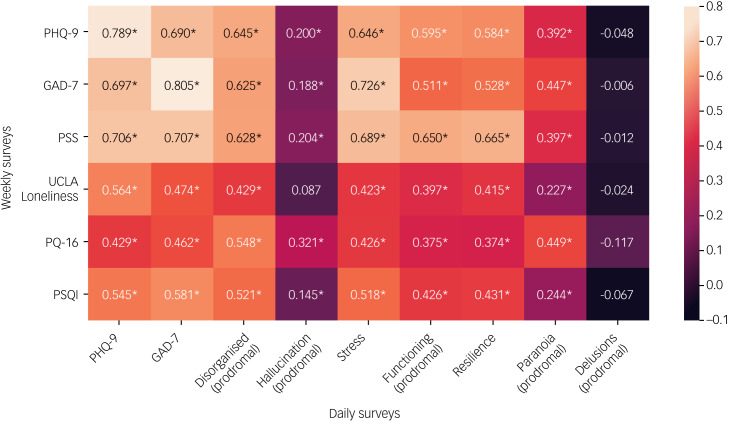
Correlations between weekly surveys and daily surveys (averaged over a given week). Correlations with *P* < 0.05 are marked with an asterisk. GAD-7, Generalised Anxiety Disorder-7; PHQ-9, Patient Health Questionnaire-9; PQ-16, Prodromal Questionnaire-16; PSQI, Pittsburgh Sleep Quality Index; PSS, Perceived Stress Scale.

**Fig. 3:**
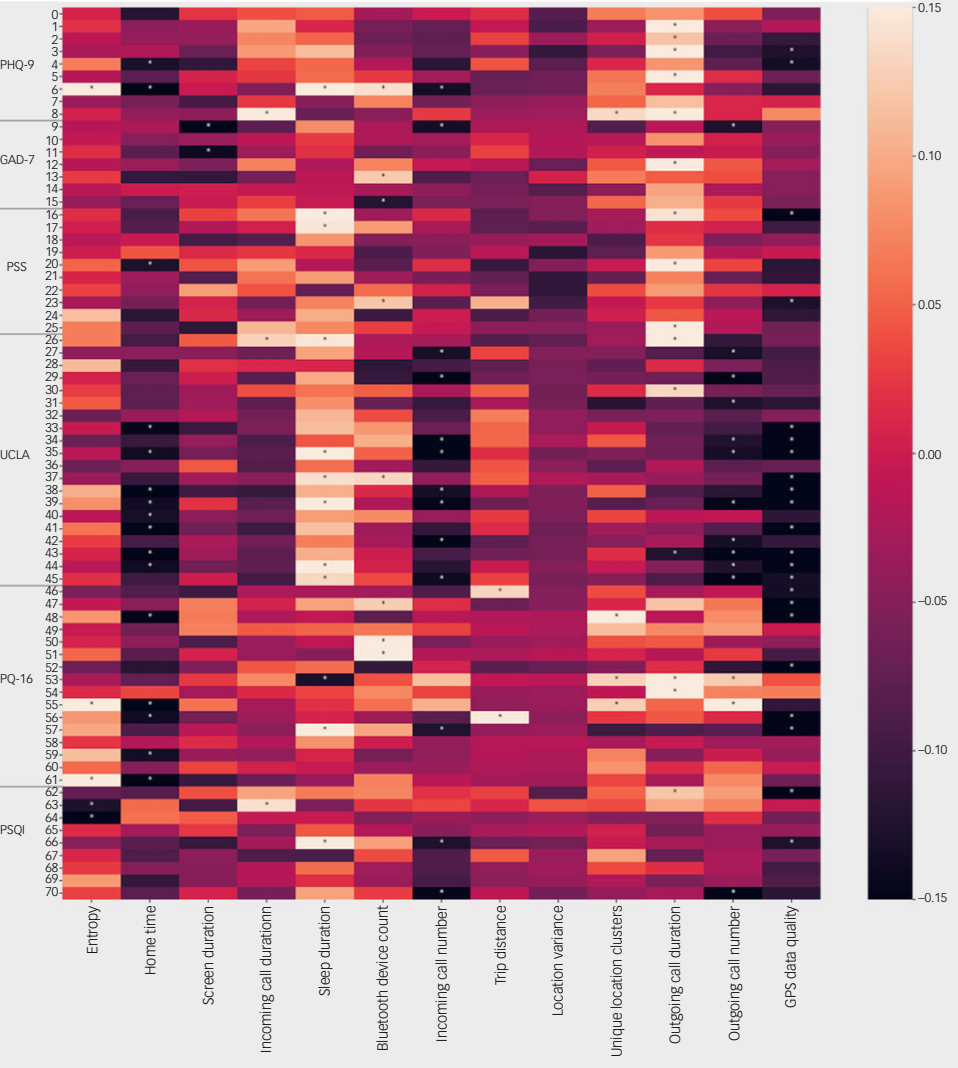
Correlations between individual survey questions and passive data features. Correlations with *P* < 0.05 are marked with an asterisk. GAD-7, Generalised Anxiety Disorder-7; PHQ-9, Patient Health Questionnaire-9; PQ-16, Prodromal Questionnaire-16; PSQI, Pittsburgh Sleep Quality Index; PSS, Perceived Stress Scale.

As daily surveys are a subset of the weekly surveys, these responses are highly correlated, as shown in Figure 2. Additionally, many of the surveys are correlated with one another. Figure 3 shows the passive features as correlated with the individual questions on each mental health questionnaire (for a full list of questions see Supplementary Appendix 1). Certain questions, such as those around loneliness, are more correlated with passive data than others.

**Fig. 4:**
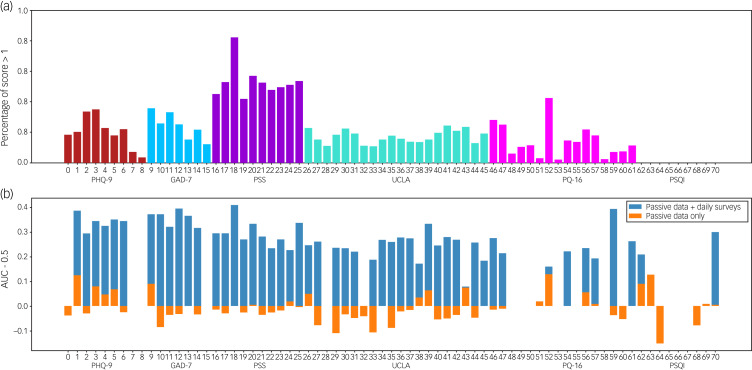
Results from the logistic regression models for each weekly survey question. (a) Percentage of weekly scores above the threshold of 1 (out of 3). (b) Results from fitting the models. The AUC is plotted with 0.5 subtracted for clarity. The model without daily surveys is shown in orange and the model with daily surveys is shown in blue. AUC, area under the curve; GAD-7, Generalised Anxiety Disorder-7; PHQ-9, Patient Health Questionnaire-9; PQ-16, Prodromal Questionnaire-16; PSQI, Pittsburgh Sleep Quality Index; PSS, Perceived Stress Scale; UCLA, UCLA Loneliness Scale.

Figure 4 shows the results of the logistic regression model trained with and without daily surveys. The model shows poor performance with only passive data. However, the daily survey models can predict weekly scores with much higher accuracy. The non-zero coefficients for models trained on passive data and daily surveys to predict total weekly scores are shown in Supplementary Appendix 2.

## Discussion

Across Figure 1, the common significant correlations indicate that we may have found a signal that does not vary based on the population of data used. Based on the results, it seems that some of the most valuable features are those derived from GPS, such as home time, information about calls and sleep duration. Screen time metrics and Bluetooth device counts do not seem to be predictive in our data-set. For some features, this may be explained by a lack of variability in the features. For example, since this data-set was collected during COVID-19, college students were likely spending more time socially distanced. As a result, the majority had limited interactions with other Bluetooth devices – the mean Bluetooth device count was 1.7. For other features, the effect of changes in passive data may be highly personalised. For example, one person may feel more comfortable at home, so greater home time may improve symptoms. For another participant increased time away from home may mean greater sociability and improved symptoms. Thus, the need to interpret this data with a patient in a shared decision-making context is critical. There may be so much variability that it is difficult to find a signal in the data, or the features may provide limited information about mental health.

The fact that some questions are more correlated with passive data than others may imply that some questions are more suited to digital mental health than others. Moreover, the results shown in Figure 4 suggest that passive data alone may not contain enough signal to predict survey scores. Short surveys on a more frequent basis can provide helpful information about a patient's state but also raise adherence concerns, as few patients will want to take surveys for extended periods of time. The fact that the regression model had non-zero coefficients for some passive features (see Supplementary Appendix 2) indicates that passive features do provide some information to enhance model predictions. It is interesting that the passive features that provide utility differ across surveys, indicating that the choice of passive features may need to be tailored to the specific survey being predicted. In the future, passive features could be used to trigger surveys as a solution to minimise adherence concerns.

### Comparison with prior work

Similar to Nickels et al,^5^ we see correlations on the order of 0.1 in Figure 1(a), lower than in prior studies.^1^ Models trained on individual survey questions also had lower AUCs than Nickels et al,^5^ which may be explained by the fact that we had a smaller sample size and a shorter study duration. Moreover, we employed different data-quality metrics than Nickels et al,^5^ which may have led to some differences. We also attempted to replicate the work of Meyerhoff et al,^6^ and our results are shown in Supplementary Appendix 3. Meyerhoff et al^6^ found correlations between the PHQ-8 and passive features, and between the Social Phobia Inventory and calls. Unlike Meyerhoff et al,^6^ we did not see significant correlations between the PHQ-9 and we did not collect data on the Social Phobia Inventory. We see significant correlations between incoming call duration, incoming call number and the number of unique location clusters and PSQI. We also see a significant correlation between incoming call duration and GAD-7.

### Limitations and future directions

Limitations of our approach include the inability to exactly replate prior studies and broader challenges inherent to this work. As we had a shorter study duration than Meyerhoff et al,^6^ it is not possible to exactly replicate that study. However, as clustering scores by groupings from *k*-means clustering is somewhat arbitrary, in this work we separated groups based on clinical phenotypes. That said, it is possible that digital phenotyping clinical correlates may not reflect current clinical concepts, but such a data-driven approach will require larger-scale studies than any mentioned in this paper. Like other studies that have struggled to replicate digital phenotyping results,^17^ we also did not capture the exact same data streams, derive the exact same features or use the exact same types of smartphones as in either Meyerhoff et al^6^ or Nickels et al.^5^ These sources of natural variation present challenges to any replication efforts, and as we showed in Figure 1(a) and 1(b), there are some differences if participants with poorer data quality are included in the analysis.

Expanding on these limitations is helpful for understanding how to design future studies and next steps for the field. First, although the sample size was large compared with other studies in this space, a larger sample size would provide a more accurate picture of college mental health. Also, criteria for inclusion in the study included elevated scores on the PSS, and as shown in Figure 4(a), only a small subset of participants showed non-zero scores on the surveys administered. Thus, it is difficult to generalise results to those with a greater degree of psychopathology. Moreover, it may be the case that we see higher correlations in participants with higher scores, such as in Figure 1(d) (of up to 0.64); however, the data-set from Figure 1(d) is small, containing only 19 weeks of data. It is difficult in small data-sets to determine if significant results are a result of the small sample size or if they truly represent a signal. Moreover, as stated above, we used different quality metrics than Nickels et al,^5^ which is a limitation because using different quality metrics (for example, requiring 75% GPS data quality rather than 50%) may cause results to vary. Currently, there is no established standard for passive data quality, and this remains an unmet need for the entire space. Given we provide the mindLAMP software free and in an open-source manner, others are at least able to replicate and advance upon our findings. In addition, since we could not collect certain data streams such as Bluetooth and call data from Androids, we did not include such data in this analysis. It is possible that differences in Android and iOS users or data collection could affect our findings. However, we note that few studies collect data from both types of devices, and Nickels et al^5^ and Meyerhoff et al^6^ both collected data from Androids only, whereas we used both Apple and Android. In addition, our study did not collect all of the same data as the other similar studies. For example, Nickels et al^5^ found that features from voice diaries and participants’ reported sleep duration had high correlations with PHQ-9 scores. These features would be interesting to explore in future work, and we have recently added this functionality into mindLAMP. Finally, as this study was run during the COVID-19 pandemic, smartphone use patterns may be different from pre-pandemic patterns. For example, as students were likely at home the majority of the time, features like Bluetooth may no longer be as useful. As other works have pointed out, mental health conditions manifest with unique patterns for each individual population-level models may be unable to separate signal from noise.^18^

In the future, we aim to use these results to further explore the utility of passive data features. Our study consisted of a population of predominantly healthy college students, using a variety of different smartphone versions and types. Although this creates a concern for validity, it also represents the challenges of real-world deployment of smartphones for mental health. We hope that future work will continue to test the validity of passive data in larger and more diverse samples. Unlike in related studies,^5,6^ since mindLAMP is an open-source platform, researchers can reproduce our study. The LAMP consortium currently consists of over 50 sites around the world, encouraging result sharing and comparisons across different populations.^9^ In addition to hand-crafted features, autoencoders could be considered for automatic feature extraction, although they require large sample sizes with less missingness to produce meaningful results. Moreover, future work should seek to train individualised models, as it may be that passive data is useful on an individual, but not population level. Although our results suggest that passive features alone may not be enough to be able to predict an individual's mental health state, these results do not dimmish its value, but rather help direct how this data can augment research efforts. When combined with short daily surveys, passive data may be able to refine and improve model predictions. We employ such a model in our digital clinic, where we use the passive data to facilitate shared decision-making between patients and clinicians.^19^

In conclusion, we investigated correlations between passive and active data features from the mindLAMP app in a large sample of college students. We found correlations lower than many previous studies, but of similar magnitude to two of the largest mental health studies to date.^5,6^ Promising features identified included those derived from GPS, anonymised smartphone call data and accelerometer-derived sleep metrics. Future work should continue to explore the utility of passive data in larger and more diverse samples. Using a combination of active and passive data to build individual models of mental illness, and recovery, for each individual offers an important next step toward applying this data to illness prevention and personalisation of care.

## Data Availability

Raw data is not available to share, but all code for the app and analysis can be found from https://docs.lamp.digital/ and linking GitHub pages.

## Supplementary material

For supplementary material accompanying this paper visit http://doi.org/10.1192/bjo.2022.507. click here to view supplementary material
