# Supplementary figures and images for: Digital phenotyping correlations in larger mental health samples: analysis and replication
Source: BJPsych Open. 2022 Jun 3;8(4):e106. doi: 10.1192/bjo.2022.507 (PMC9230632; doi:10.1192/bjo.2022.507)

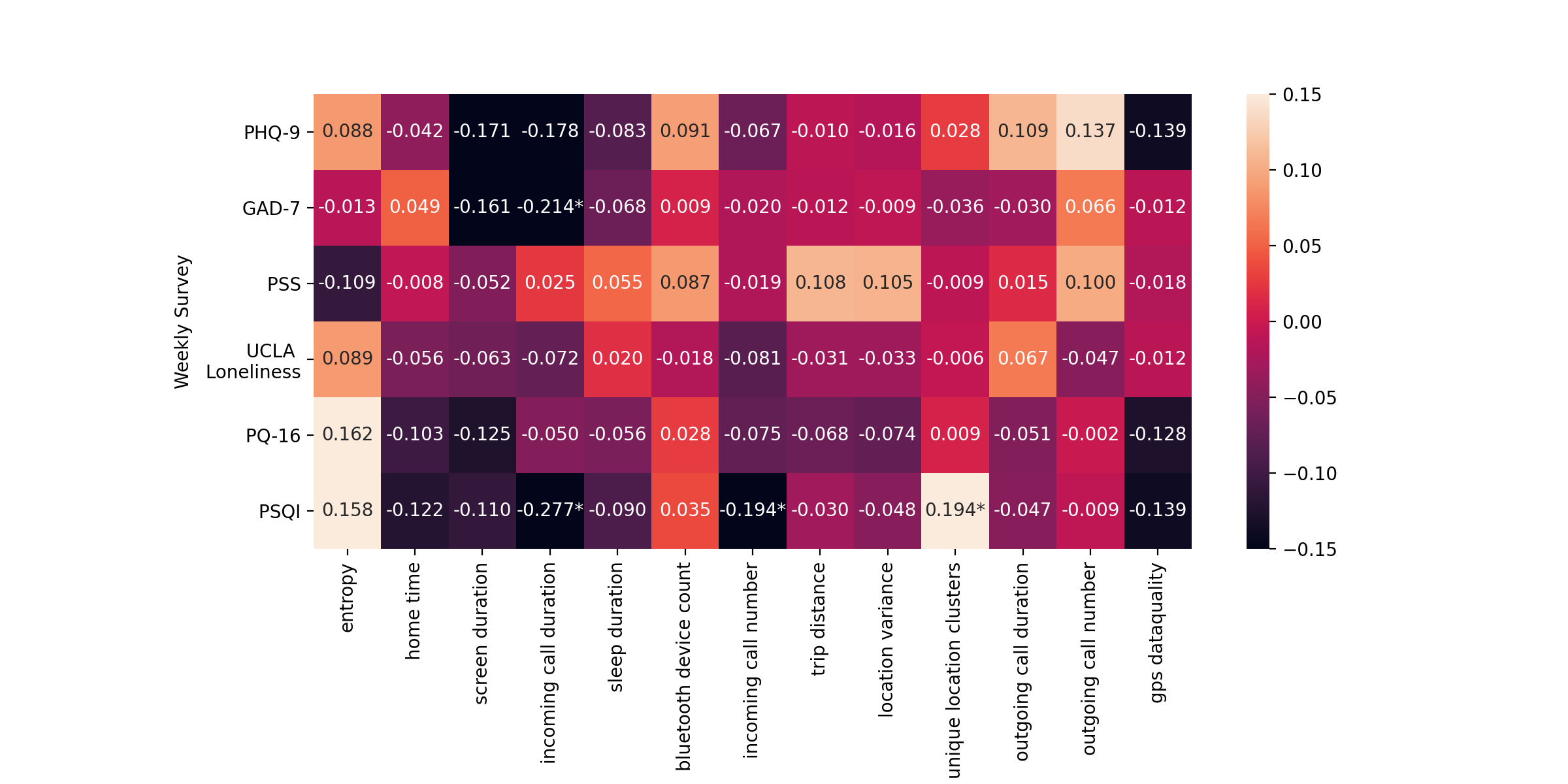

Supplement: Supplementary file 1 [file bjosup.zip › S2056472422005075sup001.tif]
